# Supplementary material for: Quantum noise and its evasion in feedback oscillators
Source: Nat Commun. 2023 Nov 4;14:7083. doi: 10.1038/s41467-023-42739-9 (PMC10625586; doi:10.1038/s41467-023-42739-9)
Supplement: Supplementary file 1 — Supplementary Information [file 41467_2023_42739_MOESM1_ESM.pdf]

Supplementary Information for "Quantum noise and its evasion in feedback oscillators"

Hudson A. Loughlin[1, *] and Vivishek Sudhir[1, 2, †]

[1] LIGO Laboratory, Massachusetts Institute of Technology, Cambridge, MA 02139
[2] Department of Mechanical Engineering, Massachusetts Institute of Technology, Cambridge, MA 02139

(Dated: October 13, 2023)

CONTENTS

I. Supplementary Note 1 — Covariance Matrices of Squeezed States 1
A. Covariance Matrices 1
B. Uncertainty Products for Entangled States 2

II. Supplementary Note 2 — Coherence Scaling with Photon Number 3

III. Supplementary Note 3 — Details of a feedback oscillator based on a phase-sensitive amplifier 5
A. Response of an oscillator with phase-sensitive amplifier 5
B. Quadrature spectra for uncorrelated in-coupled and ancillary modes 7

IV. Supplementary Note 4 — Comparison of an Ideal Purely Phase-Sensitive Oscillator to an Optical Parametric Oscillator 7

References 9

I. SUPPLEMENTARY NOTE 1 — COVARIANCE MATRICES OF SQUEEZED STATES

A. Covariance Matrices

This section defines conventions for single and two mode squeezing operators and presents expressions for the covariance matrices of squeezed and entangled states, which encode these states' quadrature spectra.

We consider the spectral covariance of modes $\{\hat{a}_i\}$ in terms of their quadratures. Defining $\hat{Q}_i \coloneqq (\hat{q}_i, \hat{p}_i)$, the the spectral covariance matrix $\boldsymbol{V}[\Omega]$ is defined by its elements [1, 2] $V_{ij}[\Omega] \coloneqq \bar{S}_{Q_i Q_j}[\Omega]$.

In our case, the modes are $\{\hat{a}_0, \hat{a}_{\mathrm{G}}\}$. When they are in vacuum states, $|\mathrm{vac}\rangle = |0\rangle_0 |0\rangle_{\mathrm{G}}$ [2]

$$\boldsymbol{V}_{\mathrm{vac}}[\Omega] = \frac{1}{2}\mathrm{diag}(1,1,1,1). \tag{1}$$

We are also interested in the state

$$|\psi\rangle \coloneqq \hat{S}_{\mathrm{E}}(r_{\mathrm{E}})\hat{S}_{\mathrm{G}}(r_{\mathrm{G}})\hat{S}_0(r_0)\,|\mathrm{vac}\rangle\,, \tag{2}$$

where

$$\begin{aligned} \hat{S}_0(r_0) &= e^{-\frac{r_0}{2}\left(\hat{a}_0^2 - \hat{a}_0^{\dagger 2}\right)} \\ \hat{S}_{\mathrm{G}}(r_{\mathrm{G}}) &= e^{-\frac{r_{\mathrm{G}}}{2}\left(\hat{a}_{\mathrm{G}}^2 - \hat{a}_{\mathrm{G}}^{\dagger 2}\right)} \\ \hat{S}_{\mathrm{E}}(r_{\mathrm{E}}) &= e^{\frac{r_{\mathrm{E}}}{2}\left(\hat{a}_0\hat{a}_{\mathrm{G}} - \hat{a}_0^\dagger \hat{a}_{\mathrm{G}}^\dagger\right)}. \end{aligned} \tag{3}$$

* hudsonl@mit.edu
† vivishek@mit.edu

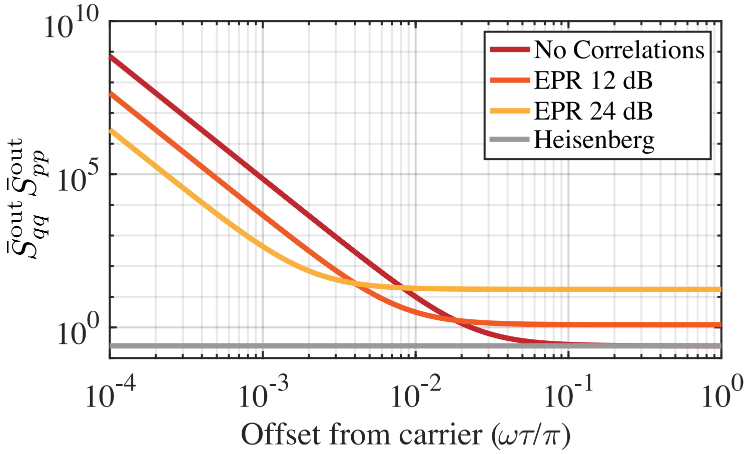

Supplementary Figure 1. **Feedback Oscillator Uncertainty Products with EPR Correlations.** Uncertainty product $\bar{S}_{qq}^{\mathrm{out}}[\Omega]\bar{S}_{pp}^{\mathrm{out}}[\Omega]$ for a feedback oscillator with phase-sensitive amplifier fed with various levels of EPR entangled states for the in-coupled and ancillary modes. As the level of squeezing used to generate entanglement is increased from zero (red) to 12 dB (orange) and then to 24dB (yellow), the uncertainty product decreases near the carrier and increases far from it. In all cases, the uncertainty product is above the Heisenberg limit (grey). Note that the uncertainty product is symmetric about the carrier.

Here, $\hat{S}_{0,\mathrm{G}}$ are single-mode squeeze operators, whose sign conventions are chosen for convenience so that for a feedback oscillator, output phase fluctuations are suppressed when $r_0 > 0$ or $r_\mathrm{G} > 0$. $\hat{S}_\mathrm{E}$ represents two mode squeezing (or EPR entanglement), and output phase fluctuations are suppressed when $r_\mathrm{E} > 0$. The covariance matrix of $|\psi\rangle$ is [2]

$$\boldsymbol{V}[\Omega] = \begin{bmatrix} \boldsymbol{I}(2r_\mathrm{G}, 2r_0, \frac{r_\mathrm{E}}{2}) & \boldsymbol{Z}(2r_0, 2r_\mathrm{G}, r_\mathrm{E}) \\ \boldsymbol{Z}(2r_0, 2r_\mathrm{G}, r_\mathrm{E})^T & \boldsymbol{I}(2r_0, 2r_\mathrm{G}, \frac{r_\mathrm{E}}{2}) \end{bmatrix}, \tag{4}$$

where we have defined the matrices $\boldsymbol{I}(x, y, z)$ and $\boldsymbol{Z}(x, y, z)$ by

$$\begin{aligned} \boldsymbol{I}(x, y, z) &= \frac{1}{2}\begin{bmatrix} e^x \sinh^2(z) + e^y \cosh^2(z) & 0 \\ 0 & e^{-x}\sinh^2(z) + e^{-y}\cosh^2(z) \end{bmatrix} \\ \boldsymbol{Z}(x, y, z) &= \frac{1}{4}\begin{bmatrix} (e^x + e^y)\sinh(z) & 0 \\ 0 & -\left(e^{-x} + e^{-y}\right)\sinh(z) \end{bmatrix}. \end{aligned} \tag{5}$$

The quadrature spectra for the two mode state with squeezed and EPR correlated modes parametrized by r_0, r_G and r_E can be read off of eq. (4).

B. Uncertainty Products for Entangled States

As mentioned in the discussion of eq. (24) in the main text, our near-resonant approximation makes it appear as if it is possible to violate Heisenberg uncertainty for sufficiently large levels of EPR entanglement between the in-coupled and ancillary modes. We now show that this is not the case.

Dropping the near-carrier approximation, the quadrature spectra of the output mode are related to those of the in-coupled and ancillary modes by

$$\begin{aligned} \bar{S}_{qq}^{\mathrm{out}}[\Omega] =& |H_0[\Omega]|^2 \bar{S}_{qq}^{0}[\Omega] + |H_\mathrm{G}[\Omega]|^2 \bar{S}_{qq}^{\mathrm{G}}[\Omega] + 2\,\mathrm{Re}\left[H_0[\Omega]H_\mathrm{G}[\Omega]^* \bar{S}_{qq}^{0,\mathrm{G}}[\Omega]\right] \\ \bar{S}_{pp}^{\mathrm{out}}[\Omega] =& |H_0[\Omega]|^2 \bar{S}_{pp}^{0}[\Omega] + |H_\mathrm{G}[\Omega]|^2 \bar{S}_{pp}^{\mathrm{G}}[\Omega] - 2\,\mathrm{Re}\left[H_0[\Omega]H_\mathrm{G}[\Omega]^* \bar{S}_{pp}^{0,\mathrm{G}}[\Omega]\right], \end{aligned} \tag{6}$$

Using the spectra from eq. (4) with $r_0 = r_\mathrm{G} = 0$, we find

$$\bar{S}_{qq}^{\mathrm{out}}[\Omega] = \frac{e^{r_\mathrm{E}}}{4\eta} + \frac{e^{-r_\mathrm{E}}\eta}{4}\left[\left(\frac{1}{\eta} - 1\right)^2 \tan^2\left(\frac{\Omega\tau}{2}\right) + 1\right] \tag{7}$$

and $\bar{S}_{pp}^{\text{out}}[\Omega] = \bar{S}_{qq}^{\text{out}}[\Omega]$. Using this expression, we find that the product $\bar{S}_{qq}^{\text{out}}[\Omega]\bar{S}_{pp}^{\text{out}}[\Omega]$ is minimized for $\Omega\tau = 2\pi n$ for $n \in \mathbb{Z}$. The minimum value of this product is

$$\min_{\Omega}\left[\bar{S}_{qq}^{\text{out}}[\Omega]\bar{S}_{pp}^{\text{out}}[\Omega]\right] = \left(\frac{e^{r_{\text{E}}}}{4\eta} + \frac{\eta e^{-r_{\text{E}}}}{4}\right)^2 \tag{8}$$

This product is itself minimized when $r_{\text{E}} = \ln(\eta)$, with the result

$$\min_{\{\Omega,\eta,r_{\text{E}}\}}\left[\bar{S}_{qq}^{\text{out}}[\Omega]\bar{S}_{pp}^{\text{out}}[\Omega]\right] = \frac{1}{4}. \tag{9}$$

Thus, for general values of Ω, η, and r_{E}, we have the relation

$$\bar{S}_{qq}^{\text{out}}[\Omega]\bar{S}_{pp}^{\text{out}}[\Omega] \geq \frac{1}{4}, \tag{10}$$

which is exactly the Heisenberg uncertainty bound. The fact that EPR entangling the in-coupled and ancillary modes does not allow the feedback oscillator to violate Heisenberg uncertainty is shown for several specific levels of squeezing in Supplementary Figure 1.

In the main text, we have seen that EPR entanglement can suppress the amplitude and phase quadratures simultaneously and to arbitrary levels in the near-resonant approximation. However, we have now shown that when we consider the full, unapproximated system dynamics, the system always obeys the Heisenberg uncertainty bound for any amount of EPR entanglement. Physically, this means that our system does not violate one of the key tenets of quantum theory, which is an important check on the validity of our results.

II. SUPPLEMENTARY NOTE 2 — COHERENCE SCALING WITH PHOTON NUMBER

Existing literature on oscillators operating below the Schawlow-Townes limit often considers oscillator stability in terms of the oscillator's coherence, C. To facilitate comparisons between this work and existing studies, we will now compute the coherence for the phase-insensitive oscillators considered in this paper.

The dimensionless coherence, C, of a feedback oscillator is defined by [3]

$$C = \max_{\omega}\left|\int_{-\infty}^{\infty}\langle\hat{a}_{\text{out}}^{\dagger}(s)\hat{a}_{\text{out}}(t)\rangle e^{-i\omega s}\right|, \tag{11}$$

and the standard quantum limit for an oscillator is taken to be $C = \mathcal{O}(\mu^2)$, where μ is the number of photons per unit time circulating in the feedback oscillator.

For an oscillator undergoing pure phase-diffusion, as is the case for the quantum noise limited phase-insensitive feedback oscillators we consider, eq. (11) is equivalent to [3]

$$C = \frac{4|\alpha|^2}{\Gamma}, \tag{12}$$

where $|\alpha|^2$ is oscillator's output photon flux and

$$\Gamma = [\omega^2/(4\pi|\alpha|^2)]\bar{S}_{pp}^{\text{out}} \tag{13}$$

is its linewidth.

For oscillators with in-coupled and ancillary modes in vacuum states, μ is related to the number of photons per unit time out-coupled from the oscillator, $|\alpha|^2$, by

$$\mu = \frac{|\alpha|^2}{1-\eta}. \tag{14}$$

Taking the output spectrum to be the Schawlow-Townes limit of eq. (16) in the main text, we find that the coherence of any phase-insensitive feedback oscillator with vacuum-state inputs is

$$C^{\text{ST}} = 16\pi\eta\tau^2\mu^2 \propto \mu^2, \tag{15}$$

the same scaling derived in [3] for the case of a laser. To use eq. (13), we have used the second-order pole approximation of the feedback oscillator's linewidth and dropped the vacuum noise term.

We now consider the coherence of feedback oscillators with entangled in-coupled and ancillary modes in the limit of high degrees of entanglement. The case with squeezed and entangled in-coupled and ancillary modes is similar. The photon flux, $\hat{N}_a$, of a field mode $\hat{a}$ is given by [4, 5]

$$\hat{N}_a(t) = \int_0^\infty \frac{d\Omega}{2\pi} \int_0^\infty \frac{d\Omega'}{2\pi} \hat{a}^\dagger[-\Omega]\hat{a}[\Omega'] e^{i(\Omega-\Omega')t}. \tag{16}$$

For a feedback oscillator with entangled modes, there is a non-zero number of photons from the entangled modes circulating in the cavity in addition to the macroscopic steady-state photon field, so the mean number of photons circulating in the oscillator is now

$$\mu = \frac{|\alpha|^2}{1-\eta} + \langle \hat{N}^-_{\rm out}(t)\rangle. \tag{17}$$

We take our frequency-domain EPR entanglement operator to be [4]

$$\hat{S}(r_{\rm E}) = \exp\left[\frac{r_{\rm E}}{2}\left(\hat{a}[\omega]\hat{b}[-\omega] - \hat{a}^\dagger[-\omega]\hat{b}^\dagger[\omega]\right)\right], \tag{18}$$

and we find

$$\begin{aligned} \hat{S}^\dagger(r_{\rm E}) a_0[\omega] \hat{S}(r_{\rm E}) &= \cosh\left[\frac{r_{\rm E}}{2}\right] a_0[\omega] - \sinh\left[\frac{r_{\rm E}}{2}\right] a^\dagger_{\rm G}[\omega] \\ \hat{S}^\dagger(r_{\rm E}) a^\dagger_{\rm G}[\omega] \hat{S}(r_{\rm E}(\omega)) &= \cosh\left[\frac{r_{\rm E}}{2}\right] a^\dagger_{\rm G}[\omega] - \sinh\left[\frac{r_{\rm E}}{2}\right] a_0[\omega]. \end{aligned} \tag{19}$$

Using the linear response equations for a phase-insensitive feedback oscillator, eq. (9) in the main text, we can write the operator for the number of photons circulating in the feedback loop, $\hat{a}^-_{\rm out}$, in terms of the in-coupled and ancillary mode operators as

$$\hat{a}^-_{\rm out}[\omega] = \frac{1}{\sqrt{1-\eta}} H_0[\omega] e^{r_{\rm E}/2}\left(\hat{a}_0[\omega] - \hat{a}^\dagger_{\rm G}[\omega]\right), \tag{20}$$

where we have assumed that we are sufficiently near resonance that we can use $H_{\rm G}[\omega] \approx -H_0[\omega]$.

For a feedback oscillator with EPR entangled modes, the in-coupled and ancillary modes are in the state $|\psi_{\rm E}\rangle = \hat{S}(r_{\rm E})|{\rm vac}\rangle$ where $|{\rm vac}\rangle \equiv |0\rangle_0|0\rangle_{\rm G}$. Using the commutator of $\hat{a}_{\rm G}$ with its adjoint, eq. (13) in the main text, we can compute

$$\langle\psi_{\rm E}|\hat{a}^{-\dagger}_{\rm out}[-\omega]\hat{a}^-_{\rm out}[\omega']|\psi_{\rm E}\rangle = \frac{|H_0[\omega]|^2 e^{r_{\rm E}}}{1-\eta}\cdot 2\pi\delta[\omega'-\omega]. \tag{21}$$

Finally, we find that the number of photons circulating in the feedback oscillator is

$$\mu = \frac{1}{1-\eta}\left(|\alpha|^2 + \frac{e^{r_{\rm E}}}{2\pi}\int_{-\Omega_0}^\infty |H_0[\omega]|^2 d\omega\right). \tag{22}$$

The integral in the second term diverges, but we can fix this issue by renormalizing the value of $|\alpha|$ and introducing a low-frequency cutoff in the squeezing parameter, $\delta\omega$. Renormalizing $|\alpha|$ is justified since any physical laser with no quantum enhancement, i.e. $r_{\rm E} = 0$, will have a finite output photon-flux, given by $|\alpha|^2$. Thus, we absorb the part of the second term with $r_{\rm E} = 0$ into α. Introducing a low-frequency entanglement cutoff is justified since physical EPR sources cannot generate entanglement between fields with zero frequency difference. Additionally, we extend the lower integral bound from $-\Omega_0$ to $-\infty$ since we are already making the near-resonance assumption that $H_{\rm G} = -H_0$. With these assumptions, the circulating photon flux is

$$\mu = \frac{1}{1-\eta}\left(|\alpha|^2 + \frac{e^{r_{\rm E}}-1}{\pi}\int_{\delta\omega}^\infty |H_0[\omega]|^2 d\omega\right), \tag{23}$$

For high levels of entanglement, such that the second term in this equation is much larger than the first and $e^{r_{\rm E}} \gg 1$, and using eq. (16) in the main text without the constant term from off-resonant vacuum noise, we have

$$\mu \approx \frac{(1/\eta - 1)}{\pi\tau^2\delta\omega} e^{r_{\rm E}} \propto e^{r_{\rm E}}, \tag{24}$$

This contrasts the Schawlow-Townes case and the case of weak entanglement where the number of photons circulating in the cavity is determined by the steady-state coherent photon number, $|\alpha|^2/(1-\eta)$, and is approximately independent of the small numbers of photons injected from the in-coupled and ancillary modes. With this expression for the circulating photon flux, we find that the output phase quadrature spectrum is given by

$$\bar{S}_{pp}^{\text{out}}[\omega] = \frac{\bar{S}_{pp}^{\text{out,ST}}[\omega]}{e^{r_{\text{E}}}} \propto \frac{\bar{S}_{pp}^{\text{out,ST}}[\omega]}{\mu}, \tag{25}$$

and the oscillator's linewidth now scales as $\Gamma \propto 1/\mu^2$. In terms of coherence, we find

$$C \propto \mu^3, \tag{26}$$

which is a parametric improvement over the Schawlow-Townes coherence limit of eq. (15).

In agreement with ref. [3, 6], we find that it is possible to design oscillators with parametrically better coherence than the Schawlow-Townes limit. We observe that ref. [3] identified a specific laser oscillator with coherence scaling as $C \propto \mu^4$, but that achieving this scaling requires that the oscillator has a nonlinear out-coupler. Our model assumes a linear out-coupler and a linearized gain medium and does not allow us to achieve quartic scaling in this manner. However, we show that it is possible to parametrically exceed the Schawlow-Townes coherence limit in any feedback oscillator, and that doing so is possible even in a linear system.

III. SUPPLEMENTARY NOTE 3 — DETAILS OF A FEEDBACK OSCILLATOR BASED ON A PHASE-SENSITIVE AMPLIFIER

This section provides extant details of the quantum noise properties of feedback oscillators with a phase-sensitive amplifier embedded in the loop.

A. Response of an oscillator with phase-sensitive amplifier

For an oscillator composed of an in-loop phase-sensitive amplifier (see Figure 1B in the main text), it is more natural to study its linear response in terms of the quadratures of the various fields involved. We define the generalized quadratures $\hat{q}_\theta$ and $\hat{p}_\theta$ by

$$\hat{q}_\theta = \frac{\hat{a}^\dagger e^{i\theta} + \hat{a}e^{-i\theta}}{\sqrt{2}}, \quad \hat{p}_\theta = \hat{q}_{\theta+\pi/2}, \tag{27}$$

which are canonically conjugate and related to the usual amplitude/phase quadratures as $\hat{q} = \hat{q}_{\theta=0}$ and $\hat{p} = \hat{p}_{\theta=0}$.

The linear response equations of eq. (25) in the main text can be expressed in terms of the quadratures. Choosing the phase angles in eq. (27) to be $\theta_{\text{out}} = \theta_{\text{out}}^+ = \theta_{\text{out}}^- = \theta_{\text{s}} = \theta_{\text{G}} = \theta_{\text{in}} = \theta_0 = \varphi_{\text{s}}/2$, the amplitude quadrature operators describing the coupled modes of a phase-sensitive oscillator satisfy

$$\begin{aligned}
\hat{q}_{\theta,\text{out}}^-[\Omega] &= e^{r_{\text{s}}}\hat{q}_{\theta,\text{s}}[\Omega] \\
\hat{q}_{\theta,\text{s}}[\Omega] &= G\,\hat{q}_{\theta,\text{in}}[\Omega] + \sqrt{G^2-1}\,\hat{q}_{\theta,\text{G}}[\Omega] \\
\hat{q}_{\theta,\text{out}}^+[\Omega] &= -\sqrt{\eta}\,\hat{q}_{\theta,\text{out}}^-[\Omega] + \sqrt{1-\eta}\,\hat{q}_{\theta,0}[\Omega] \\
\hat{q}_{\theta,\text{out}}[\Omega] &= \sqrt{1-\eta}\,\hat{q}_{\theta,\text{out}}^-[\Omega] + \sqrt{\eta}\,\hat{q}_{\theta,0}[\Omega] \\
\hat{q}_{\theta,\text{in}}[\Omega] &= e^{i\Omega\tau}\hat{q}_{\theta,\text{out}}^+[\Omega],
\end{aligned} \tag{28}$$

and the phase quadrature operators for this oscillator satisfy

$$\begin{aligned}
\hat{p}_{\theta,\text{out}}^-[\Omega] &= e^{-r_{\text{s}}}\hat{p}_{\theta,\text{s}}[\Omega] \\
\hat{p}_{\theta,\text{s}}[\Omega] &= G\,\hat{p}_{\theta,\text{in}}[\Omega] - \sqrt{G^2-1}\,\hat{p}_{\theta,\text{G}}[\Omega] \\
\hat{p}_{\theta,\text{out}}^+[\Omega] &= -\sqrt{\eta}\,\hat{p}_{\theta,\text{out}}^-[\Omega] + \sqrt{1-\eta}\,\hat{p}_{\theta,0}[\Omega] \\
\hat{p}_{\theta,\text{out}}[\Omega] &= \sqrt{1-\eta}\,\hat{p}_{\theta,\text{out}}^-[\Omega] + \sqrt{\eta}\,\hat{p}_{\theta,0}[\Omega] \\
\hat{p}_{\theta,\text{in}}[\Omega] &= e^{i\Omega\tau}\hat{p}_{\theta,\text{out}}^+[\Omega].
\end{aligned} \tag{29}$$

We note that unlike the phase-insensitive amplifier case where all phase angles were relative to the oscillator's output phase θ_{out}, the phase angles are now all defined relative to the squeeze angle φ_{s}. Having made this point, we will now take $\varphi_{\text{s}} = 0$.

We now define the $\hat{q}$ and $\hat{p}$ quadrature transfer functions $H_0^q[\Omega]$, $H_{\text{G}}^q[\Omega]$, $H_0^p[\Omega]$, and $H_{\text{G}}^p[\Omega]$ by

$$\begin{aligned}\hat{q}_{\text{out}}[\Omega] &= H_0^q[\Omega]\hat{q}_0[\Omega] + H_{\text{G}}^q[\Omega]\hat{q}_{\text{G}}[\Omega] \\ \hat{p}_{\text{out}}[\Omega] &= H_0^p[\Omega]\hat{p}_0[\Omega] - H_{\text{G}}^p[\Omega]\hat{p}_{\text{G}}[\Omega].\end{aligned} \tag{30}$$

The sign convention is chosen such that $H_0^q = H_0^p = H_0$ and $H_{\text{G}}^q = H_{\text{G}}^p = H_{\text{G}}$ in the case that $r_{\text{s}} \to 0$ and the phase-sensitive amplifier reduces to a phase-insensitive amplifier.

With a phase-sensitive amplifier in the feedback loop, the saturation condition of eq. (42) in the main text is modified. Still modeling the phase-sensitive amplifier as a phase-insensitive amplifier and a squeezer, the phase-insensitive amplifier will saturate when the loop gain for the amplified quadrature is equal to unity. Mathematically, this condition is

$$Ge^{r_{\text{s}}}\sqrt{\eta} = 1, \tag{31}$$

which tells us that in steady state, we have $G = e^{-r_{\text{s}}}/\sqrt{\eta}$. If the amplifier is purely phase-sensitive, and thus represented by an ideal squeezer, then $G = 1$, and $e^{r_{\text{s}}} = 1/\sqrt{\eta}$. We denote this value of r_{s} by r_{max}. For squeezing above this value, saturation effects will reduce r_{s} back to r_{max}. Explicitly, r_{max} is given by

$$r_{\text{max}} \equiv -\frac{1}{2}\ln(\eta). \tag{32}$$

Solving eqs. (28) and (29) and using the condition of eq. (31) to eliminate G from these equations, we find that the quadrature transfer functions are given by

$$\begin{aligned}H_0^q[\Omega] &= \frac{e^{i\Omega\tau}/\sqrt{\eta} + \sqrt{\eta}}{1 + e^{i\Omega\tau}} = H_0[\Omega] \\ H_{\text{G}}^q[\Omega] &= \frac{\sqrt{1/\eta - e^{2r_{\text{s}}}}\sqrt{1-\eta}}{1 + e^{i\Omega\tau}} = \sqrt{\frac{1-\eta e^{2r_{\text{s}}}}{1-\eta}}H_{\text{G}}[\Omega] \\ H_0^p[\Omega] &= \frac{e^{i\Omega\tau}/\sqrt{\eta} + e^{2r_{\text{s}}}\sqrt{\eta}}{e^{2r_{\text{s}}} + e^{i\Omega\tau}} \\ H_{\text{G}}^p[\Omega] &= \frac{\sqrt{1/\eta - e^{2r_{\text{s}}}}\sqrt{1-\eta}}{e^{2r_{\text{s}}} + e^{i\Omega\tau}}.\end{aligned} \tag{33}$$

From these equations, we see that $H_{\text{G}}^q[\Omega] \to 0$ and $H_{\text{G}}^p[\Omega] \to 0$ as $r \to r_{\text{max}}$. Physically, the amplifier does not add any noise to the output as it becomes completely phase-sensitive.

We see that near resonance where $\Omega\tau \approx (2n+1)\pi$, H_0^q and H_{G}^q still scale as $1/\omega$, whereas H_0^p and H_{G}^p have different denominators which do not approach zero as ω becomes small.

The spectra of the output amplitude and phase are related to those of the in-coupled and ancillary modes as:

$$\bar{S}_{qq}^{\text{out}}[\Omega] = |H_0^q[\Omega]|^2\bar{S}_{qq}^0[\Omega] + |H_{\text{G}}^q[\Omega]|^2\bar{S}_{qq}^{\text{G}}[\Omega] + 2\text{Re}\left[H_0^q[\Omega]H_{\text{G}}^q[\Omega]^*\bar{S}_{qq}^{0,G}[\Omega]\right], \tag{34}$$

and

$$\bar{S}_{pp}^{\text{out}}[\Omega] = |H_0^p[\Omega]|^2\bar{S}_{pp}^0[\Omega] + |H_{\text{G}}^p[\Omega]|^2\bar{S}_{pp}^{\text{G}}[\Omega] - 2\text{Re}\left[H_0^p[\Omega]H_{\text{G}}^p[\Omega]^*\bar{S}_{pp}^{0,G}[\Omega]\right]. \tag{35}$$

From eqs. (33) and (34) we see that the output amplitude quadrature of the phase-sensitive oscillator retains the same spectral shape as in the phase-insensitive oscillator, determined by $|H[\Omega]|$. However, as the oscillator becomes more phase-sensitive, its amplifier contributes less noise to the output amplitude quadrature.

Further, from eqs. (33) and (35), we see that the output phase-quadrature spectrum of the phase-sensitive oscillator has a fundamentally different spectral shape than it does for a phase-insensitive oscillator. The phase-quadrature transfer functions no longer have poles at $\omega\tau = 0$.

Using the fact that $\hat{a}_0$ and $\hat{a}_{\text{out}}$ are freely propagating bosonic modes and the transfer functions from eq. (33), we can compute the statistics of the amplifier's ancillary mode. As in the case of the phase-insensitive amplifier, $\hat{a}_{\text{G}}$ is not freely propagating, so its statistics need not be bosonic. Computing the ancillary mode's statistics, we find

$$[\hat{a}_{\text{G}}[\Omega], \hat{a}_{\text{G}}^\dagger[\Omega']] = 2\pi \cdot \delta[\Omega + \Omega'], \tag{36}$$

so as for a phase-insensitive feedback oscillator, the amplifier's ancillary mode obeys bosonic statistics despite the fact that it is an in-loop field.

B. Quadrature spectra for uncorrelated in-coupled and ancillary modes

In the absence of correlations between the in-coupled and ancillary modes, eqs. (34) and (35) reduce to

$$\begin{aligned}\bar{S}^{\text{out}}_{qq}[\Omega] &= |H^q_0[\Omega]|^2 \bar{S}^0_{qq}[\Omega] + |H^q_{\text{G}}[\Omega]|^2 \bar{S}^{\text{G}}_{qq}[\Omega] \\ \bar{S}^{\text{out}}_{pp}[\Omega] &= |H^p_0[\Omega]|^2 \bar{S}^0_{pp}[\Omega] + |H^p_{\text{G}}[\Omega]|^2 \bar{S}^{\text{G}}_{pp}[\Omega].\end{aligned} \tag{37}$$

So the amplitude and phase quadrature spectra of the phase-sensitive feedback oscillator with uncorrelated input modes are given by

$$\begin{aligned}\bar{S}^{\text{out}}_{qq}[\omega] &= \left(1 + \frac{(\sqrt{\eta} - 1/\sqrt{\eta})^2}{4\cos^2(\Omega\tau/2)}\right) \bar{S}^0_{qq}[\Omega] + \left(\frac{(1/\eta - e^{2r_{\text{s}}})(1-\eta)}{4\cos^2(\Omega\tau/2)}\right) \bar{S}^{\text{G}}_{qq}[\Omega] \\ \bar{S}^{\text{out}}_{pp}[\omega] &= \left(\frac{\left(e^{r_{\text{s}}}\sqrt{\eta} - \frac{1}{e^{r_{\text{s}}}\sqrt{\eta}}\right)^2 + 4\cos^2(\Omega\tau/2)}{4\sinh^2(r_{\text{s}}) + 4\cos^2(\Omega\tau/2)}\right) \bar{S}^0_{pp}[\Omega] + \left(\frac{\left(1 - 1/(\eta e^{2r_{\text{s}}})\right)(1-\eta)}{4\sinh^2(r_{\text{s}}) + 4\cos^2(\Omega\tau/2)}\right) \bar{S}^{\text{G}}_{pp}[\Omega].\end{aligned} \tag{38}$$

IV. SUPPLEMENTARY NOTE 4 — COMPARISON OF AN IDEAL PURELY PHASE-SENSITIVE OSCILLATOR TO AN OPTICAL PARAMETRIC OSCILLATOR

We compare the model of an ideal, purely phase-sensitive oscillator developed in the main text and Supplementary Note III to the dynamics of an optical parametric oscillator (OPO). We consider a degenerate OPO with a signal field in mode $\hat{a}$ and a pump field in mode $\hat{b}$. We take the OPO's interaction Hamiltonian to be $H_{\text{int}} = i\hbar\chi(\hat{a}^{\dagger 2}\hat{b} - \hat{a}^2\hat{b}^\dagger)$, and assume the cavity decay rates for the signal and pump fields are given by γ_a and γ_b respectively.

Given these assumptions, an OPO is governed in the Markovian limit by the input-output equations [7–9]

$$\begin{aligned}\partial_t \hat{a} &= -\gamma_a \hat{a} + 2\chi \hat{b}\hat{a}^\dagger + \sqrt{2\gamma_a}\hat{a}_0 \\ \partial_t \hat{b} &= -\gamma_b \hat{b} - \chi \hat{a}^2 + \sqrt{2\gamma_b}\hat{b}_{\text{in}}\end{aligned} \tag{39}$$

and the boundary condition

$$\hat{a}_{\text{out}} = \sqrt{2\gamma_a}\hat{a} - \hat{a}_0, \tag{40}$$

where we have called input signal mode $\hat{a}_0$ in line with the naming conventions in the main text.

We now solve these equations in expectation value to find the steady-state mean output field amplitude in the signal mode, $\hat{a}$. For mode $\hat{a}$ ($\hat{b}$), we denote the mean field amplitude by $\bar{a}$ ($\bar{b}$). As in a typical OPO, we take the input signal mode, $\hat{a}_0$ to be vacuum and the input pump mode to be a coherent state with amplitude $\bar{b}_{\text{in}}$. In steady-state, $\partial_t \bar{a} = \partial_t \bar{b} = 0$ and we find [9]

$$\begin{aligned}\bar{a} &= \frac{\gamma_a \gamma_b}{2\chi^2}(\sigma - 1) \\ \bar{b} &= \frac{\gamma_a}{2\chi}\end{aligned} \tag{41}$$

where σ is defined to be

$$\sigma \equiv 2\sqrt{\frac{2\chi^2}{\gamma_a^2\gamma_b}}\bar{b}_{\text{in}}, \tag{42}$$

and determines whether or not the OPO is above threshold. We have chosen phases such that $\bar{a}$, $\bar{b}$, and $\bar{b}_{\text{in}}$ are real-valued. For $\sigma < 1$, the oscillator is below threshold and has no macroscopic output in its signal mode. In this case, the OPO is referred to as an optical parametric amplifier (OPA). For $\sigma > 1$, the OPO is above threshold and

has a macroscopic output in its signal mode. Above threshold, the output level in the signal beam is governed by pump depletion, and given mathematically by eq. (41).

Following ref. [9], we now linearize eq. (39) about the mean dynamics given by eq. (41). Defining $\hat{a} = \bar{a} + \delta\hat{a}$ and similarly for the other modes, we find that the system's fluctuations are given by the coupled equations

$$\begin{aligned} \partial_t \delta\hat{a} &= -\gamma_a \delta\hat{a} + \gamma_a \delta\hat{a}^\dagger + \sqrt{2\gamma_a\gamma_b(\sigma-1)}\delta\hat{b} + \sqrt{2\gamma_a}\delta\hat{a}_0 \\ \partial_t \delta\hat{b} &= -\gamma_b \delta\hat{b} - \sqrt{2\gamma_a\gamma_b(\sigma-1)}\delta\hat{a} + \sqrt{2\gamma_b}\delta\hat{b}_{\text{in}} \end{aligned} \tag{43}$$

For the remainder of this section, we will focus on the fluctuations and denote $\delta\hat{a}$ by $\hat{a}$ etc. Fourier transforming, and writing eq. (43) in terms of quadrature operators, we find

$$\begin{aligned} -i\Omega\hat{q}_a[\Omega] =& \sqrt{2\gamma_a\gamma_b(\sigma-1)}\hat{q}_b[\Omega] + \sqrt{2\gamma_a}\hat{q}_0[\Omega] \\ -i\Omega\hat{q}_b[\Omega] =& -\gamma_b\hat{q}_b[\Omega] - \sqrt{2\gamma_a\gamma_b(\sigma-1)}\hat{q}_a[\Omega] + \sqrt{2\gamma_b}\hat{q}_b^{\text{in}}[\Omega] \end{aligned} \tag{44}$$

for the amplitude quadrature and

$$\begin{aligned} -i\Omega\hat{p}_a[\Omega] =& -2\gamma_a\hat{p}_a[\Omega] + \sqrt{2\gamma_a\gamma_b(\sigma-1)}\hat{p}_b[\Omega] + \sqrt{2\gamma_a}\hat{p}_0[\Omega] \\ -i\Omega\hat{p}_b[\Omega] =& -\gamma_b\hat{p}_b[\Omega] - \sqrt{2\gamma_a\gamma_b(\sigma-1)}\hat{p}_a[\Omega] + \sqrt{2\gamma_b}\hat{p}_b^{\text{in}}[\Omega] \end{aligned} \tag{45}$$

for the phase quadrature.

Using eqs. (44) and (45) and the boundary condition of eq. (40) to solve for the output amplitude and phase quadratures,we find

$$\begin{aligned} \hat{q}_{\text{out}}[\Omega] &= \left(\frac{2\sqrt{2(\sigma-1)}\gamma_a\gamma_b}{2(\sigma-1)\gamma_a\gamma_b + (-i\gamma_b-\Omega)\Omega}\right)\hat{q}_b^{\text{in}}[\Omega] + \left(\frac{2\gamma_a(i\Omega-\gamma_b)}{2(\sigma-1)\gamma_a\gamma_b + (-i\gamma_b-\Omega)\Omega} - 1\right)\hat{q}_0[\Omega] \\ \hat{p}_{\text{out}}[\Omega] &= \left(\frac{2\sqrt{2(\sigma-1)}\gamma_a\gamma_b}{2\gamma_a\gamma_b\sigma - i(2\gamma_a+\gamma_b)\Omega - \Omega^2}\right)\hat{p}_b^{\text{in}}[\Omega] + \left(\frac{-2(\sigma-1)\gamma_a\gamma_b + \Omega(i\gamma_b+\Omega)}{2\gamma_a\gamma_b\sigma - i(2\gamma_a+\gamma_b)\Omega - \Omega^2}\right)\hat{p}_0[\Omega]. \end{aligned} \tag{46}$$

In eq. (46), the $\hat{q}_b^{\text{in}}$ and $\hat{p}_b^{\text{in}}$ terms represent pump noise, and the other terms represent noise from the input vacuum signal mode coupled to the output.

We will now show how eq. (46) reduces to eq. (26) in the main text in the appropriate limit. First, we take the limit that $\gamma_b, \chi \to 0$ with χ^2/γ_b held constant. Physically, this is the limit that the pump photons have an extremely long lifetime in the feedback loop while keeping the output photon flux in the signal mode held constant. In this limit, eq. (46) reduces to

$$\begin{aligned} \hat{q}_{\text{out}}[\Omega] &= \left(\frac{2i\gamma_a}{\Omega} - 1\right)\hat{q}_0[\Omega] \\ \hat{p}_{\text{out}}[\Omega] &= \left(\frac{i\Omega}{2\gamma_a - i\Omega}\right)\hat{p}_0[\Omega] \end{aligned} \tag{47}$$

From eq. (47), we can read off the relations between the OPO's input and output spectra. We have

$$\begin{aligned} \bar{S}_{qq}^{\text{out,a}}[\Omega] &= \left(1 + \frac{4\gamma_a^2}{\Omega^2}\right)\bar{S}_{qq}^0[\Omega] \\ \bar{S}_{pp}^{\text{out,a}}[\Omega] &= \left(\frac{\Omega^2}{4\gamma_a^2 + \Omega^2}\right)\bar{S}_{pp}^0[\Omega], \end{aligned} \tag{48}$$

which agree with the output spectra for an ideal, purely phase-sensitive oscillator, given by eq. (26) in the main text, near resonance. Near resonance, $\cos^2(\Omega\tau/2) \approx \Omega^2\tau^2/4$, and the amplitude decay rate is given by $\gamma_a = (1/\sqrt{\eta} - \sqrt{\eta})/(2\tau)$. We note that the input-output equations of eq. (39) assume the system has a single resonance at $\Omega = 0$. To recover the series of resonances described by eq. (26) in the main text, Ω in eq. (48) can be replaced by $\omega = \Omega_0 + \Omega$.

We have seen that the idealized behavior of a purely phase-sensitive oscillator, given by eq. (26) in the main text, arises as the limiting case of an OPO in which the pump mode has a very long lifetime in the resonator, the nonlinear interaction is weak, and we consider the quadrature spectra near resonance. In this model, pump depletion

leads to amplifier saturation and the system's equations of motion can be appropriately linearized about the system's steady-state dynamics.

---

[1] R. Simon, N. Mukunda, and B. Dutta, Physical Review A **49**, 1567 (1994).
[2] S. L. Braunstein and P. van Loock, Reviews of Modern Physics **77**, 513 (2005).
[3] T. J. Baker, S. N. Saadatmand, D. W. Berry, and H. M. Wiseman, Nature Physics **17**, 179 (2020).
[4] K. J. Blow, R. Loudon, S. J. D. Phoenix, and T. J. Shepherd, Phys. Rev. A **42**, 4102 (1990).
[5] S. L. Danilishin and F. Y. Khalili, Living Reviews in Relativity **15**, 5 (2012).
[6] C. Liu, M. Mucci, X. Cao, M. V. G. Dutt, M. Hatridge, and D. Pekker, Nature Communications **12**, 5620 (2021).
[7] M. J. Collett and C. W. Gardiner, Physical Review A **30**, 1386 (1984).
[8] C. W. Gardiner and M. J. Collett, Physical Review A **31**, 3761 (1985).
[9] C. Fabre, E. Giacobino, A. Heidmann, and S. Reynaud, Journal de Physique **50**, 1209 (1989).
